# Robust features for the automatic identification of autism spectrum disorder in children

**DOI:** 10.1186/1866-1955-6-12

**Published:** 2014-05-23

**Authors:** Justin Eldridge, Alison E Lane, Mikhail Belkin, Simon Dennis

**Affiliations:** 1 Department of Computer Science and Engineering, The Ohio State University, 395 Dreese Laboratories, 2015 Neil Avenue, Columbus OH, USA; 2 School of Health Sciences, University of Newcastle, University Drive, Callaghan NSW 2308, Australia; 3 School of Psychology, University of Newcastle, University Drive, Callaghan NSW 2308, Australia

**Keywords:** Autism spectrum disorder, Event-related potential, EEG, Classification

## Abstract

**Background:**

It is commonly reported that children with autism spectrum disorder (ASD) exhibit hyper-reactivity or hypo-reactivity to sensory stimuli. Electroencephalography (EEG) is commonly used to study neural sensory reactivity, suggesting that statistical analysis of EEG recordings is a potential means of automatic classification of the disorder. EEG recordings taken from children, however, are frequently contaminated with large amounts of noise, making analysis difficult. In this paper, we present a method for the automatic extraction of noise-robust EEG features, which serve to quantify neural sensory reactivity. We show the efficacy of a system for the classification of ASD using these features.

**Methods:**

An oddball paradigm was used to elicit event-related potentials from a group of 19 ASD children and 30 typically developing children. EEG recordings were taken and robust features were extracted. A support vector machine, logistic regression, and a naive Bayes classifier were used to classify the children as having ASD or being typically developing.

**Results:**

A classification accuracy of 79% was achieved, making our method competitive with other automatic diagnosis methods based on EEG. Additionally, we found that classification performance is reduced if eye blink artifacts are removed during preprocessing.

**Conclusions:**

This study shows that robust EEG features that quantify neural sensory reactivity are useful for the classification of ASD. We showed that noise-robust features are crucial for our analysis, and observe that traditional preprocessing methods may lead to poor classification performance in the face of a large amount of noise. Further exploration of alternative preprocessing methods is warranted.

## Background

Autism spectrum disorder (ASD) is a lifelong neurodevelopmental disorder of increasing prevalence and community concern [1]. Individuals with ASD experience difficulties with social communication and their behavior is often stereotypic and repetitive. The symptoms of the disorder vary in severity between individuals but may necessitate support services to facilitate social engagement, classroom learning and independent living in adulthood [2]. It is commonly reported that children with ASD exhibit hyper-reactivity or hypo-reactivity to sensory stimuli [3]. In particular, children with ASD have been known to display extreme distress to loud or unexpected noises, or to under-react by failing to orient to their name or respond to painful stimuli [4-6]. Recently, parent-reported behaviors associated with sensory reactivity have been successfully used to classify meaningful subgroups of children with ASD [7-9]. Such reports suggest that electrophysiological measurements of sensory reactivity may be a useful biomarker for ASD classification in children.

Event-related potentials (ERPs) are used to examine the sensory function of children with ASD, particularly in the auditory domain [10,11]. ERPs are observed using electroencephalogram (EEG) recordings of stimuli-driven changes over time in the electrical activity of the cortex of the brain. ERPs can be measured using the polarity, amplitude, latency, and scalp distribution of the potentials due to neural activation. Paradigms for ERP studies vary depending on the sensory modality and mechanism of impairment of interest. A common paradigm used in the ASD ERP literature is the oddball paradigm [10]. This paradigm, in which a ‘standard’ stimulus is presented most of the time (e.g. 85%) and a ‘deviant’ or ‘novel’ stimulus is presented less frequently (e.g. 15%), is employed when investigating sensory novelty discrimination and may be considered a measure of neural sensory reactivity. A number of authors have reported differences in the ERP profiles of individuals with ASD compared with typically developing (TD) controls in response to an auditory oddball paradigm. Specifically, for more complex auditory stimuli such as speech sounds, individuals with ASD are observed to display delayed or absent orientation to novel stimuli. Attenuated responses are noted both in early auditory processing (vis-à-vis brainstem responses) and later processing (vis-à-vis mismatch negativity, P3) [10,11]. It is hypothesized that these differences may underlie behavioral deficits in ASD including non-attention to name being called (due to attenuated responses to speech stimuli) and social communication impairments (due to failure to integrate and attend to higher level speech stimuli). Measurement by EEG, however, is an inherently noisy process, which is made worse by the artifacts introduced by young subjects and those with functional difficulties such as those commonly observed in ASD. Attempts to classify individuals with ASD using EEG data are hampered by the abundance of noise in the recordings.

Much of the recent work in the classification of ASD from EEG data has focused on the spectral coherence of EEG signals. Spectral coherence measures the consistency of the phase difference between two EEG signals over time [12]. Numerous studies have shown significant differences in coherence between ASD and typical subjects [13-15]. A recent study by Duffy and Als [12] involving a large sample of 1,304 subjects aged 1 through 18 years demonstrated high classification accuracy when spectral coherence features were used. High classification rates of around 90% persisted when the sample was limited to smaller age ranges. Similarly, Bosl *et al*. [16] used machine-learning methods to classify EEG signals as either ASD or typical. The study used the modified multiscale entropy of the EEG signal to measure the non-linear complexity of brain activity. A high classification accuracy (70 to 90%) of boys with ASD was demonstrated, with near perfect accuracy obtained for boys aged 9 months.

Much of the recent literature involving EEG studies takes a traditional approach to artifact rejection, in which data is removed on a per epoch basis. The textbook by Luck [17] lists several common measures used to reject epochs, including simple threshold rejection in which an epoch is removed if it contains data outside of a specified range. With such methods, the researcher must carefully choose a rejection criterion: making the criterion too strict will result in many non-artifacts being removed from the data, while being too lenient will leave much of the noise intact [18]. A common criterion used to judge the viability of an epoch asserts that all channels be artifact free. As the number of electrodes in the sensor array increases, so does the probability of rejecting an epoch on any given channel. Junghöfer *et al*. [19] recognized this phenomenon and proposed a statistical approach to artifact rejection, in which artifactual sensors are interpolated rather than discarded to retain the maximum amount of data in the presence of noise.

It is also standard practice to remove certain types of artifacts, such as those introduced by eye blinks, using blind source separation by independent component analysis (ICA) [20]. In this approach, it is assumed that the data recorded at each of the *N* electrodes is a linear combination of *N* independent sources. The goal of ICA is to ‘unmix’ the data recorded at the electrodes into the temporally independent source components. Ideally, one or several of these components will correspond to eye blink events. Such components can be identified by strong activity at the periocular electrode sites. Eye blink removal is accomplished by removing these components from the data. Previous literature, however, has identified that eye blink rate is a marker of central dopaminergic activity with links to general arousal levels [21], and one study has reported that eye blink rate is elevated in autism [22]. Further, increased eye blink rate is associated with anxiety and engagement in speech and memory tasks [21]. Reduced eye blink rates have been observed in attention deficit hyperactivity disorder and intellectual disability [21-23]. Removal of eye blink data as recommended in standard artifact rejection processes, therefore, may inadvertently conceal a salient marker of neural differences specific to ASD.

The aims of the study were twofold. First, we developed a novel approach to the generation of EEG features useful for the classification of ASD in young children where the degree of noise is substantial. Two aspects of this approach are emphasized: 1) the rejection of artifactual data in the face of extreme noise and 2) the construction of robust features. We propose an amendment to the traditional artifact removal pipeline that allows for the retention of greater amounts of data while using comparatively strict rejection criteria. Second, we tested an automated classification framework based on the robust features developed for the first aim and assessed the accuracy of the framework against other recently reported methods. We evaluated the performance of this classification framework in two conditions: with and without eye blink data.

## Methods

### Participants

Participants for the study were children aged 6 to 10 years with a diagnosis of ASD (*n*=19) and TD same-aged peers (*n*=30). Inclusion criteria for the ASD participants were: 1) ASD diagnosis had been made by a qualified practitioner using standard diagnostic measures; 2) participant was able to cooperate with all study procedures; 3) hearing and middle ear function was assessed as intact (described below); and 4) no history of other neurological or seizure disorder. Where possible, all ASD participants were further assessed by members of the research team to ascertain ASD symptom severity using standard measures (described below). ASD symptom severity data for three ASD participants were unavailable due to non-attendance at the scheduled clinical evaluation appointment. Inclusion criteria for the TD participants were that: 1) participant was able to cooperate with all study procedures; 2) hearing and middle ear function was assessed as intact (see below); and 3) participant had no history of language delay, learning disability, or neurological, seizure or auditory processing disorder. ASD participants were recruited to the study via flyers and announcements distributed through parent and professional networks, autism clinics and schools in the Central Ohio area, and private therapy practices. Typically developing participants were recruited via ResearchMatch, a web-based volunteer registry [24], broadcast emails to faculty and staff at The Ohio State University, and personal contacts. Approvals for this project were gained from the Institutional Review Board at The Ohio State University, protocol number 2009B0418. Table 1 details the characteristics of the participants included in the study.

**Table 1:** Participant characteristics

**Group**	***n***	**Mean age in**	**Gender**	**ASD symptom severity**
		**years (SD)**		
ASD	19	8.46 (1.3)	*M*=16, *F*=3	*ADOS* (*n*=13):
				Mean CSS = 6.23 (SD = 2.31)
				*CARS Severity Rating* (*n*=6):
				Non-autistic: *n*=1
				Mild/moderate: *n*=2
				Severe: *n*=3
TD	30	8.17 (1.26)	*M*=15, *F*=15	N/A

Two participants received both the CARS and ADOS; two participants did not receive either the CARS or ADOS. ADOS, Autism Diagnostic Observation Schedule; ASD, autism spectrum disorder; CARS, Childhood Autism Rating Scale (second edition); CSS, Calibrated Severity Score; N/A, not applicable; SD, standard deviation; TD, typically developing.

### Procedure

Participants in the study were involved in a larger study examining the neural profiles of children with ASD classified using parent-reported sensory features. ASD symptoms were assessed in our study using either the Autism Diagnostic Observation Schedule (ADOS) [25] or the Childhood Autism Rating Scale (second edition) (CARS2) [26]. The ADOS was administered by a qualified clinical psychologist to characterize ASD symptoms. Four different modules of the ADOS are available for use depending on the expressive language skills of the child. Modules 1 to 3 were used for this study; these modules consist of 10 to 14 guided activities in which the child’s behaviors are observed in response to a particular scenario or staged task (e.g. free play, bubble play, snack and anticipation of a social routine). A detailed behavioral coding guide is then provided for completion based on observations made throughout the entire evaluation. Behaviors coded relate to the core diagnostic criteria for ASD including language and communication, reciprocal social interaction, play, stereotyped behaviors, restricted interests and other abnormal behaviors. Behaviors are rated on a scale of 0 to 3 based on the rate and intensity of the behavior observed. Cut-off scores are provided in the ADOS to determine the likelihood that the child has an ASD. In this study, the Calibrated Severity Score (CSS) was used to determine ASD symptom severity.

The CARS2 was administered by the second author to participants who were unable to attend an ADOS session. The CARS2 is a 15-item behavior rating scale, which is used to diagnose and differentiate children with ASD from TD children and other developmentally disabled children. Behavioral items assessed by the CARS2 include relating to people; imitation; emotional response; body use; object use; adaptation to change; visual response; listening response; taste, smell and touch response and use; fear/nervousness; verbal communication; non-verbal communication; activity level; level and consistency of intellectual response; and general impressions. Each item is scored on a four-point scale; total scores range from 15 to 60. The total scores on the CARS2 are used to distinguish the child’s placement on the ASD spectrum (non-autistic, mild/moderate ASD, or severe ASD). The reliability of the CARS ranges in the literature from alpha coefficients of 0.73 to 0.94 [27,28]. In [29], Eaves and Milner found that the CARS had a sensitivity of 98%.

Hearing and middle ear function was assessed prior to the ERP protocol by research assistants trained by an audiologist using standard audiometry. Participants who failed the hearing screen were referred to an audiologist for follow-up testing. All participants included in the study were assessed to have adequate hearing for the ERP protocol.

The ERP protocol was as follows: 

1. *Apparatus*: Data were collected using an EGI GES 300 system (Electrical Geodesics, Inc, Eugene, OR) utilizing a HydroCel 128 Channel Geodesic Sensor Net and a Net Amps 300 amplifier. The Cz electrode was used as the reference electrode.

2. *Auditory paradigm*: Speech sounds were presented using an oddball paradigm in blocks of 400 stimuli, each block lasting approximately 8.5 minutes. The standard (repetitive) stimuli were presented 85% of the time and deviant (novel) stimuli presented 15% of the time. The stimulus duration was 340 ms and the inter-stimulus interval was 960 ms. Synthesized speech was utilized, specifically phonemes (*dae* and *daa*). Phonemes were chosen as previous studies have reported differences in speech sound processing between ASD and typical controls [10]. For each block, which phonemes were chosen as the standard and deviant stimuli was random. The order of presentation of each phoneme was pseudo-randomized. Within each block, at least four standard stimuli were presented before and after each deviant stimulus.

3. *Data collection procedure*: Continuous EEG data were collected in a small, soundproof room with auditory stimuli presented binaurally via wall speakers. The ERP recording device was in a room adjacent to the data collection room. During the presentation of the auditory stimuli, a caregiver and/or research assistant remained in the room with the participant. Participants watched a silent movie or played on an iPad throughout the protocol. iPad games were preselected by the research team and only those that were found to generate minimal movement were offered to the participant during data collection. iPad games were also played without sound. Movement breaks were offered to participants between blocks of trials to increase compliance with the protocol and maximize data collected.

### Artifact removal

The data were preprocessed to remove artifacts. First, baselining was performed by subtracting the mean of the 200 ms of the pre-stimulus signal. Next, artifacts were rejected on a channel-by-epoch basis, in which the rejection of data on a given channel and epoch was independent of data taken on other channels during the same epoch. That is, data from epoch *e* and channel *c* may be rejected while data from epoch *e*, channel *c*^′^ is kept. This method of rejecting data on a channel-by-epoch basis differs from the more traditional approach in which data from all channels and a given epoch is discarded, even if only data from one of the channels is found to be artifactual.

More specifically, the raw data from a single subject before rejection can be expressed as the three-dimensional array $X\in {\mathbb{R}}^{C\times E\times T}$, with *C*, *E*, and *T* indicating the number of channels, epochs, and time samples, respectively. As described above, across the study the number of channels *C* was 128, and the number of time samples *T* was 300. The number of recorded epochs *E* differed between subjects, but was typically around 1,000. Traditional rejection amounts to selecting a principal subarray of *X*, obtained by striking out rows and columns corresponding to noisy channels and epochs. That is, Ĉ channels are first rejected across the study, leaving a data array of shape (C-Ĉ)×E×T. After this, Ê noisy epochs are rejected, leaving a data array of shape (C-Ĉ)×(E-Ê)×T. This is the method described in [17].

On the other hand, the result of our approach was not a rectangular array, as a different number of epochs were rejected on each channel. Rather, our technique was to form a set of pairs *P*={(*c*,*e*)} for each subject, such that if (*c*,*e*)∈*P*, then epoch *e* on channel *c* is ‘clean’, i.e., not rejected. The remaining data were then the set of vectors *D*={*x*_*c*,*e*,1:*T*_:(*c*,*e*)∈*P*}. In this notation, 1:*T* denotes that the elements from 1 to *T* are selected, so that *x*_*c*,*e*,1:*T*_ is a vector in ${\mathbb{R}}^{T}$ representing all time samples from channel *c* and epoch *e*. In the following analysis, we often deal with the data from standard and deviant epochs separately. That is, we form the sets *S*={*x*_*c*,*e*,1:*T*_:(*c*,*e*)∈*P* and *e* is a standard epoch} and *D*={*x*_*c*,*e*,1:*T*_:(*c*,*e*)∈*P* and *e* is a deviant epoch}. As such, while traditional artifact rejection amounts to the removal of entire epochs, our approach removed individual epoch-channels.

In what follows, two strategies were used: threshold and trend-line rejection. In threshold rejection, an epoch-channel was removed if a portion of the raw signal deviated by ±100 *μ*V from the baseline. In trend-line rejection, a line was fitted to the signal and the epoch-channel was rejected if the slope of the line exceeded 50 *μ*V per epoch with a minimum coefficient of determination of 0.3. An epoch-channel was discarded if it was rejected by either threshold or trend-line rejection, or both.

**Figure 1:**
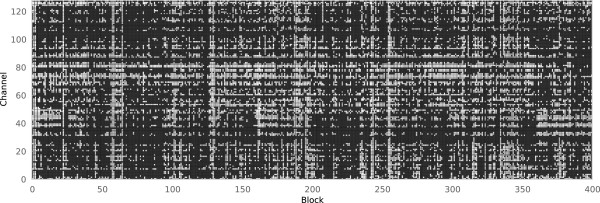
**Channel rejection rates.** Rejected epoch-channels from a block of 400 epochs. All 400 epochs were recorded from the same individual during one session. A white cell indicates that the epoch-channel was rejected by threshold or trend-line rejection, while a black cell was kept.

Figure 1 shows the result of our approach to rejection on a single block of 400 epochs, using the threshold and trend-line rejection parameters described above. If the (*i*,*j*) pixel is white, the data from the *i*th channel and the *j*th epoch were rejected. After the rejection process, the data remaining were from the black region of the image. For the *j*th epoch to be considered clean using the traditional approach to rejection, the entire *j*th column of the image would need to be black. As a result, the traditional approach rejections all of the epochs of this block, as each epoch is marked for rejection on at least one channel. On the other hand, out of the 51,200 epochchannel slices in the data shown in Figure 1, our method rejects 15,430. Therefore, roughly 70% of the data from the block were retained.

This departure from the standard was done in response to the large amount of noise in the data. To demonstrate this, the following experiment was performed: noisy channels were rejected on a per block basis by computing the kurtosis of the observations on a given channel across all epochs. The channel was rejected if the kurtosis was greater than five standard deviations away from zero. This resulted in an average of eight channels being rejected per block. Next, epochs were rejected using simple threshold rejection with several thresholds, ranging from 100 *μ*V to 1,000 *μ*V.

**Figure 2:**
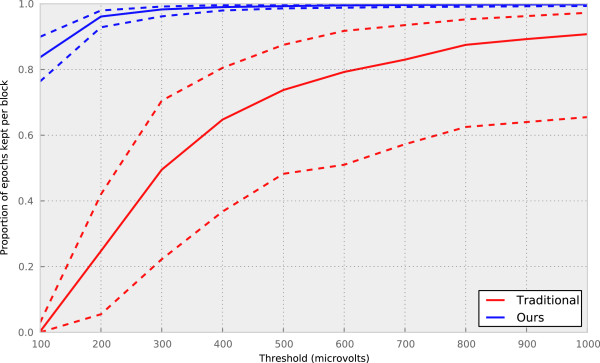
**Comparison of rejection methods.** The proportion of epochs retained per block is shown as a function of the threshold, for both our method (blue) and the traditional approach to rejection (red). The solid line shows the median number of epochs rejected per block, and the dashed lines show the upper and lower quartiles.

The median proportion of epochs kept per block as a function of the threshold is shown by the solid red line in Figure 2, along with the upper and lower quartiles (dashed lines). Mostly notably, if the rejection threshold is set at 100 *μ*V, none of the data is kept. If the objective were to keep, on average, 75% of the data from any given block, the threshold would need to be raised to nearly 500 *μ*V. This threshold is several times the suggestion given in [17], and will likely result in the inclusion of many obvious outlying artifacts.

Alternatively, our approach of rejecting individual epoch-channels as opposed to entire epochs drastically reduces the amount of data rejected, as shown by the blue lines in Figure 2. Most strikingly, at the commonly used threshold of 100 *μ*V, 85% of the data are kept on average, whereas none of the data would be kept using the traditional approach. This method allows for more stringent rejection thresholds than commonly used in more general EEG studies, and for the inherently noisier data collected from young children, without catastrophic rejection rates.

Lastly, it should be noted that while it is common to clean the data of eye blink artifacts by, for example, rejecting characteristic ICA components, this approach was not taken in this analysis. Rather, we found that including eye blink artifacts is an important part of the classification process. This result is further discussed below (see ‘The importance of eye blinks and artifact removal’).

### Features

#### *Sum of signed differences*

The ERP study was designed to examine the difference between ERPs in ASD and typical children. In an associated study, Lane *et al.* (under review) found that ASD and typical children can be characterized by the difference between their responses to deviant stimuli and to standard stimuli. In particular, it was found that in children with ASD, the deviant evoked potential was significantly higher than the standard evoked potential at certain key times of the response. This difference between the deviant and standard responses was not significant in typical children.

**Figure 3:**
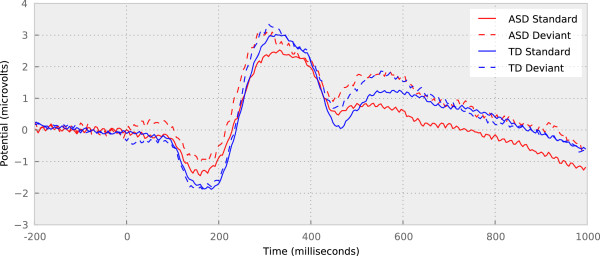
**Median waveforms for each condition and stimulus type.** Note the magnitude of the difference between the ASD standard response and deviant between 0 and 200 ms, and from 400 ms onwards. This difference is not as pronounced in the typical waveforms. ASD, autism spectrum disorder; TD, typically developing.

Figure 3 shows the median standard and deviant waveforms for both TD and ASD children, averaged over the entire study, and using the artifact rejection approach described previously. Notably, the response to deviant stimuli by ASD participants was more positive than their response to standard stimuli, particularly from 0 to 200 ms and from 400 ms onwards. To test the significance of this difference, the sum of signed differences (SSD) was computed in four intervals (0 to 150 ms, 150 to 250 ms, 250 to 400 ms, and 400 to 1,000 ms) and for each subject. A *t*-test was then used to test the null hypothesis that the SSD of ASD subjects and the SSD of typical subjects were drawn from normal distributions with the same mean. The results of the *t*-tests are shown in Table 2. In the interval from 0 to 150 ms and in the interval from 400 to 1,000 ms, the distributions are significantly different at the *P*=0.05 level, and in the interval from 150 to 250 ms the difference is marginally significant.

**Table 2:** Significance of difference between conditions of SSD in various intervals

**Time interval (ms)**	***P* value**
0 – 150	0.0020
150 – 250	0.0905
250 – 400	0.3148
400 – end	0.0118

SSD, sum of signed differences.

To quantify this observed difference, the median standard and deviant responses were calculated for each subject. In terms of the notation used in the ‘Artifact removal’ section above, this involved the calculation of the median vectors of the set of standard responses *S* and the set of deviant responses *D*. Recall that *S* and *D* are sets of vectors in ${\mathbb{R}}^{T}$, where *T* is the number of time samples recorded per epoch. We calculated the median standard response $\bar{s}\in {\mathbb{R}}^{T}$ in a straightforward manner: by finding the element-wise median of the vectors in *S*. That is, the *i*th element of $\bar{s}$ is the median of all of the *i*th elements of vectors in *S*: ${\bar{s}}_{i}=\text{median}\{x_{i}:x\in S\}$. The median deviant response $\bar{d}$ was calculated in an analogous fashion.

To measure the difference between a subject’s average standard response and average deviant response in a more suitable manner for a classification algorithm, we used the SSD in various time intervals of the response waveform. The SSD was computed by subtracting the median standard response from the median deviant response at each time sample, yielding a 1,200 ms difference waveform. This was partitioned into 24 equal-length segments, each corresponding to a 50 ms interval of signal. The area under the difference curve was found in each region, giving 24 SSD features representing a single subject. Note that the median waveforms were used here, as opposed to the mean waveforms, as the median is more robust to outliers.

More explicitly, given the typical standard waveform $\bar{s}$ and the typical deviant waveform $\bar{d}$, both vectors in ${\mathbb{R}}^{T}$, the difference vector $\delta =\bar{d}-\bar{s}$ was computed. The SSD in the *i*th region was then found by integrating:

$$\overset{50i}{\underset{j=50(i-1)+1}{\sum }}\delta_{j}$$

The SSD statistic quantifies the difference between the deviant and standard responses. A positive SSD suggests that the deviant response is higher than the standard response in a region. Furthermore, because the SSD combines information across time samples, it further reduces the effect of noise.

#### *Variance in time*

In addition to the SSD statistic, the variance of the typical standard waveform was computed for use as a feature. Given the difference waveform *δ* as computed above, the variance was computed in 12 partitions:

$$v_{i}=\mathrm{var}\{\delta_{j}|100(i-1)+1\leq j\leq 100i\}$$

This represents the variability of the typical response over time. Note that the variance was computed in fewer partitions (12) than the SSD feature (24). This was done so that each partition was larger, thereby including more data for each variance calculation.

#### *Modified multiscale entropy*

The procedure outlined by [16] was used to compute the modified multiscale entropy (mMSE) feature for each subject. The mMSE was calculated over 20 s of data gathered by concatenating several 1.2 s epochs, for a total of 5,000 contiguous time samples. Every other channel was discarded so that only 64 electrodes were considered, and 20 timescales were used in the calculation. These choices for the number of time samples, electrodes, and timescales match those used by [16].

### Classification

Before classification, the features above were combined in three different ways: 

1) Our features: First, we combined the SSD features with the variance features. Recall that the SSD was computed in 24 partitions, each corresponding to 50 ms of signal. Because the first 200 ms of the response was taken pre-stimulus presentation, the first four partitions were discarded, leaving 20 SSD features.

A small but statistically significant group difference was found in the variance of the standard response shortly after stimulus presentation. The fourth, fifth, and sixth partitions, corresponding to the variance between 200 ms and 400 ms post-stimulus, were kept, leaving three variance features. The 20 SSD features and three variance features were concatenated into a 23-dimensional feature vector. 

2) mMSE: The mMSE features described by [16] were calculated as described above, resulting in a 1,280-dimensional feature vector for each subject.

3) Our features + mMSE classification. A linear support vector machine (SVM) was trained with penalty parameter *C*=0.1, and evaluated on each subject using leave-one-out cross-validation (LOOCV). The resulting classification for each subject was appended to our feature vector to produce a 24-dimensional feature vector for each participant.

The decision to use the SVM’s prediction rather than concatenating the mMSE features with ours directly was made because of the disparity in dimensionality between the two feature sets. The mMSE features vastly outnumber the SSD and variance features, and so directly concatenating the two would tend to produce a classifier focused on the features from [16].

An alternative approach is to use the predictions of classifiers trained on our features and the features from [16] separately, producing a two-dimensional feature vector. This method was tested, but generally resulted in poorer classification accuracy than using the approach outlined above, and is therefore omitted from the discussion below. Each set of features was used with several machine-learning algorithms implemented in the Scikit-Learn machine-learning package [30]. For an overview of each classifier, see the textbook by Theodoridis and Koutroumbas [31].

#### *Support vector machine*

A linear SVM was trained on each of the three feature configurations. The performance of the classifier was evaluated by LOOCV. A nested threefold cross-validation process was used to pick the penalty parameter *C*. In this process, a training fold produced by LOOCV was split in three, and the performance of a parameter selection was evaluated by training on two of the sets and testing on the third, then permuting the training sets and averaging the accuracy over all permutations. A logarithmic interval of [ 2^-4^,2^6^] was searched over to obtain the optimal *C*. Because there were more typical participants in the study, the class weights were adjusted so that the weighting of each example was inversely proportional to the class’s frequency in the data.

#### *Logistic regression*

An l2-regularized logistic regression classifier was trained on each of the data sets. An l1-regularized classifier was also tested, though found to perform worse and therefore omitted from the following presentation. The performance of the classifier was evaluated using LOOCV, and the regularization parameter *C* was chosen using nested threefold cross-validation over the interval [ 2^-5^,2^5^].

#### *Naive Bayes*

A Gaussian naive Bayes classifier was trained on each of the configurations. Given an example, the result of the classifier is the probability estimate of the example belonging to the typical class, conditioned on the data. To account for the unbalanced data set, this probability estimate was compared to a threshold chosen by nested LOOCV to produce a final, hard classification label. The performance of the classifier was evaluated using LOOCV.

## Results

Table 3 shows the accuracy obtained for different classifiers on the three feature sets. The accuracy is weighted so that each class contributes equally. More specifically, if there are *n*_1_ examples in class 1 and *n*_-1_ examples in class 2, and *c*_1_,*c*_-1_ are the number of correctly classified examples in each class, the weighted accuracy is

$$\frac{n_{-1}c_{1}+n_{1}c_{-1}}{2n_{1}n_{-1}}$$

**Table 3:** Classifier performance for various feature sets

**Classifier**	**Our features**	**mMSE**	**Ours + mMSE**
SVM	0.69	**0.69**	0.67
Logistic regression	0.67	0.63	0.63
Naive Bayes	**0.77**	0.60	**0.79**

The best performance for each set is in bold. Numbers shown denote the accuracy of the classifier. mMSE, modified multiscale entropy; SVM, support vector machine.

The naive Bayes classifier resulted in the best performance of 79% accuracy when our features were combined with those from [16]. To determine the statistical significance of the classification accuracy, the data labels were permuted 100 times and the accuracy of the classifier recorded for each permutation. This was done for the naive Bayes classifier using only our features, and for the naive Bayes classifier using the stacked features. In both cases every permutation resulted in a lower classification accuracy than that shown above. Additionally, the sensitivity of the classifier was computed to be 0.68 and the specificity to 0.87. Reports of precision for current clinical diagnostic measures range from a sensitivity of 0.91 to 0.98 and a specificity of 0.5 to 0.84 [32].

### The importance of eye blinks and artifact removal

As previously mentioned, eye blink artifacts were not removed before analysis. It is interesting to note that the classification result above is contingent upon the eye blinks being left in the data. Table 4 shows the result of using the same features and classifier hyperparameters as used in the previous analysis, but with removing eye blink artifacts after epoch rejection. More specifically, eye blink artifacts were removed by ICA. Principal component analysis was used to decompose each block of data into ten components, which were then further decomposed into independent components. Up to two components resembling eye blink artifacts were manually identified and subtracted.

**Table 4:** Classifier performance with eye blinks removed

**Classifier**	**Our features**	**mMSE**	**Ours + mMSE**
SVM	0.45	0.52	0.41
Logistic regression	0.58	0.58	0.54
Naive Bayes	0.42	0.56	0.43

Numbers denote the accuracy of the classifier. mMSE, modified multiscale entropy; SVM, support vector machine.

**Figure 4:**
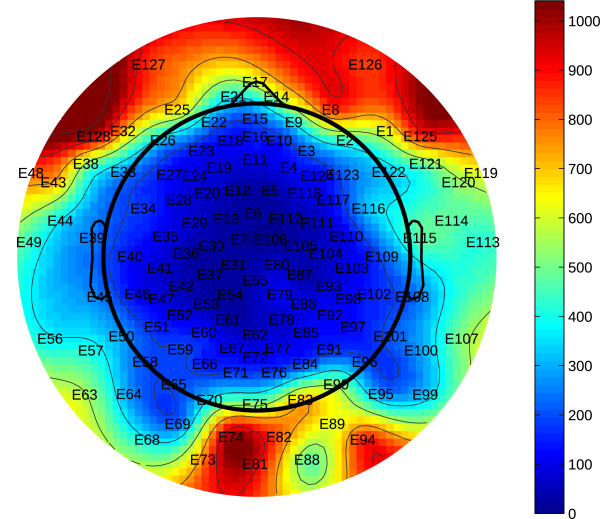
**Electrode rejection rates.** Topographical plot showing the number of epochs rejected for each electrode across the entire study. The electrodes at the perimeter of the sensor net suffered the most rejections.

As can be seen, each classifier performs at chance. This result suggests that the inclusion of eye blink artifacts may be useful for the classification of ASD by EEG. This hypothesis is further supported by group differences in the number of epochs rejected from the front electrodes. The topographical plot in Figure 4 shows how the number of rejected epochs varies across electrodes. More epochs were rejected from the electrodes on the perimeter of the net compared to electrodes in the center. This is likely due to the difficulty in obtaining a clean contact between the extreme electrodes and the comparably irregular surfaces on the side, rear, and front of the head. It can also be seen that the electrodes with the most rejected epochs were placed on the front of the scalp. This is to be expected, as these electrodes are those most affected by eye blink artifacts.

Table 5 shows the median proportion of epochs rejected for several electrodes placed on the front of the scalp for both ASD and TD individuals. The result of a Kruskal–Wallis test provides the accompanying *P* value. On all but one electrode, the median proportion of epochs rejected from ASD subjects was significantly higher than the median proportion rejected from their neurotypical counterparts. This suggests that the data from the ASD subjects are noisier for these electrodes. One simple hypothesis is that the frequency of eye blinks under the presentation of novel auditory stimuli differs between ASD and typical children.

**Table 5:** Rejection rates for selected periorbital channels, ASD and TD

**Channel**	**ASD**	**TD**	***P* value**
127	69%	61%	0.05
17	58%	49%	0.02
126	67%	57%	0.08
21	55%	47%	0.05
14	53%	54%	0.21
25	70%	60%	0.03
8	69%	61%	0.02

The *P* value measures the significance of the difference between the rejection rate in ASD subjects and the rate in TD subjects. ASD, autism spectrum disorder; TD, typically developing.

The above results suggest that the performance of our classifier is driven largely by differences in the frequency of eye blinks between ASD and neurotypical subjects. If this is the case, what is the utility of using EEG in classification? That is, if our method draws largely from disparities in ocular artifacts, could a simple count of eye blinks achieve a similarly high classification accuracy? To answer these questions, eye blink artifacts were counted automatically. ICA components were computed as described above. For each subject, the component most resembling an eye blink artifact was manually selected. The activation of this component was zero-meaned across the subject’s data, and the standard deviation was computed. The standard deviation was used to set a subject-specific threshold: if the activation of the eye blink component in a given epoch exceeded this threshold, the epoch was marked as containing an eye blink. In this manner, eye blink counts were accumulated for every subject in the study. Using a subject’s eye blink frequency as his or her sole representative feature, a weighted resubstitution accuracy of 66% was obtained, compared to the 79% accuracy obtained by our classifier in cross-validation.

A classifier using eye blink counts alone therefore appears to be weakly predictive. While our classifier appears to draw much of its classification performance from the frequency of eye blinks, it is nevertheless able to obtain a significantly higher accuracy, thereby vindicating the use of the more complicated EEG data. This suggests that the removal of eye blink components has the unintended side effect of removing a signal that contains information that is predictive of autism.

### A note on gender

Given that the set of ASD subjects and neurotypical subjects had very different ratios of males to females, it is possible that the classifier was biased so that it was classifying on the basis of gender, not condition. For example, if the classifier labeled all males as ASD, and all females as neurotypical, an unweighted accuracy of nearly 75% would be obtained.

To address this concern, only the data from the male subjects was used in training and testing, leaving 16 ASD subjects and 15 typical subjects. If the classifier were in fact a gender predictor, it would achieve near-chance accuracy on this data set. In fact, the naive Bayes classifier yielded 74% accuracy, which is sufficiently close to the 79% accuracy of the classifier on the full data set to conclude that the effect of the gender bias is small, if existent.

## Discussion

The purpose of this study was to develop and test a novel approach to the generation of EEG features useful in the classification of ASD. Our approach was to develop the SSD statistic as a robust measure of a subject’s neural sensory reactivity. Our results suggest that this method is competitive with other previously reported approaches, achieved accepted sensitivity and specificity, and was unaffected by gender bias. Of most importance, however, was the fact that this result was achieved on a small data set with data retention optimized in preprocessing.

Any analysis of EEG data must address the large amount of noise inherent in the process. Many methods exist to clean EEG data of eye blink and muscle artifacts and electrode noise. Commonly used approaches include the removal of ICA components that are typical of eye blinks, rejection of noisy channels by kurtosis, and epoch rejection by threshold or trend-line fitting. Oftentimes these methods cannot be automated or require the expertise of an EEG technician, making them difficult to reproduce.

It has also been seen, both in the above findings and by others [12], that the amount of data discarded as noise by artifact removal techniques may be influenced by the condition that we wish to diagnose. In the case of our analysis, the inclusion of artifactual data was an integral part of our method; removing these ‘artifacts’ (vis-à-vis eye blinks) significantly reduced classification performance.

This emphasizes the importance of artifact rejection in the process of building a classifier. The process can be described with three steps: 

1. Reject artifactual data.

2. Compute features on the cleaned data.

3. Learn a classifier using these features.

A common approach is to remove as much noise as possible in the first step so as to compute features from the data that are believed to be more representative of the underlying brain activity. One downside of this approach is that clean EEG data are often difficult to distinguish from artifactual data, particularly for young participants or those with neurobehavioral disorders [12]. As a result, it is not always clear whether the data being removed are noise, or whether they reflect brain activity that may in fact be predictive of the underlying condition. As the complexity of an artifact rejection method increases, the interpretation of what data are being removed often becomes more difficult, compounding the problem. Another downside of this approach is that it is particularly devastating to studies involving younger subjects, who are more likely to introduce additional artifacts by fidgeting or through non-cooperation. Adopting an aggressive artifact rejection approach leaves little remaining data, which increases the danger of overfitting.

Our approach is to spread the burden of artifact reduction over all three steps listed above. First, we adopt very simple, automated rejection methods to preprocess the data. The goal in this step is not to remove all artifacts, merely those which are obviously noise. We argue that our simple approach to artifact rejection is easier to implement, understand, and reproduce. It also has the benefit of retaining much more data.

It is likely the case that the retention of these data, some of which would be considered artifactual by traditional approaches to rejection, results in more noise in the rest of the pipeline. For this reason, we use a small set of robust features to reduce noise further. In the above analysis, we favored the median over the mean wherever possible, as the former is more robust to outliers. This averaging allows us to combine as much information as possible to reduce noise to the fullest extent. For example, the typical standard response was found by gathering all of a subject’s responses to standard stimuli, regardless of channel or epoch, then taking the median at each sample time across all responses. As a result, nearly 100,000 measurements were used to compute each sample point of the typical standard waveform for each subject. By incorporating as much data as possible into these grand averages, we extend artifact rejection in the preprocessing stage to artifact suppression in the feature-building stage.

EEG studies often involve a small number of participants, with the control group outnumbering the group of interest. This is also the case in our study. Overfitting is a supreme danger in such cases, even if much care is taken to preprocess the data and to keep the feature set small and robust to noise. We found that the naive Bayes classifier was the most capable of handling the small, unbalanced samples of this study. A possible reason for this success is the independence assumption of the classifier; features are assumed to be independent of one another. For this reason, the classifier is less likely to fit interactions between the features. In reality, the naive Bayes assumption of feature independence is most likely incorrect, but learning these subtle interactions requires much more training data than are often available.

In [33], Hand *et al.* give another possible explanation for the simpler naive Bayes classifier outperforming the more sophisticated models. In particular, they argue that simpler methods may outperform more complicated approaches when the distinction between classes is not clearly defined. In these cases, class labels include a large amount of arbitrariness, and more complicated models learn idiosyncrasies of the design data, which the simpler models cannot, essentially leading to overfitting. As ASD is, famously, a *spectrum*, the boundary between neurotypical and ASD subjects is not clearly defined, and so Hand *et al.*’s discussion is appropriate.

Our approach for rejecting epoch-channels can be interpreted as a weighting of the channels based on how noisy they are. Each channel contributes a different number of epochs to the study, with noisier channels contributing fewer epochs. Consider an especially noisy channel, in which only a few epochs are deemed to be clean with respect to our rejection criteria. These epochs may still contain useful information, and we would therefore like to include them in the average waveforms computed for the subject. On the other hand, we might be skeptical of the data from this channel, given that such a large proportion of its data is artifactual. This skepticism is implicitly considered by the process of averaging, as a channel’s contribution to the final average is dependent upon how many unrejected epochs it contains, i.e., how noisy it is. This probabilistic interpretation is an advantage over methods depending on the interpolation of channels.

The traditional approach does not enjoy this benefit, as each channel contributes the same number of epochs to the grand average. If, as in our study, the rejection criteria must be made very permissive to retain a reasonable amount of data, it is quite likely that the epochs kept, and therefore the channels kept, vary significantly in the amount of noise they contain. For example, consider a channel that contains many epochs that are close to our (now very lenient) rejection criteria but still deemed to be non-artifactual. This channel will contribute just as much data to the grand average as a channel for which all epochs are far away from being considered artifactual. This is in contrast to our approach: we can make the rejection criteria much tighter, therefore decreasing the variability among the kept epoch-channels.

Further, the results of our study reveal that eye blink activity during an auditory oddball paradigm contributes strongly to the accuracy of our classification framework. Standard ERP protocols remove eye blink data on the assumption that they are purely ‘artifactual’. As a measure of central dopaminergic activity with links to general arousal, however, eye blink data may enhance the discrimination power of ERP techniques. Further investigation of eye blink rate in concert with other measures of sensory reactivity is warranted to explore its utility as a marker of salient neural differences in ASD.

## Conclusions

In this paper we have shown the competitiveness of a framework for classifying ASD in young children using EEG data collected from an auditory oddball paradigm. Central to our framework is the twofold approach of simple artifact rejection on a channel-by-epoch basis and the construction of robust features. In particular, we have found that features that quantify the difference in response to novel auditory stimuli (SSD), combined with the mMSE features used by [16], give the best classification performance.

We have provided evidence that traditional artifact rejection approaches may be ill suited for EEG studies involving children. Most notably, the amount of noise inherent in such studies combined with the increased probability of a channel containing noise, introduced by high-density sensor arrays, leads to devastating rejection rates for usually sensible rejection criteria. In response to this, we discard the usual assertion that for a given epoch to be clean, it must be clean on every channel. Instead, we reject individual epoch-channel slices. We found that this allows for the retention of much more data.

It may be argued that performing artifact rejection in this fashion leads to more noise in the remainder of the classification pipeline. We addressed this issue by using robust features and simple classification algorithms that are resistant to overfitting. Furthermore, we presented evidence that artifact rejection for the sake of artifact rejection might be detrimental to classification performance. Most notably, we showed that the inclusion of eye blink artifacts leads to improved classification accuracy.

In conclusion, we note that an objectively superior method of comprehensive artifact rejection that applies to every EEG study likely does not exist. Therefore, while traditional approaches and rules of thumb may apply to many studies, special consideration is necessary in cases involving unusual amounts of noise, such as studies involving children with ASD. In this paper, we have presented evidence that a streamlined approach to artifact rejection combined with robust features leads to the highest classification accuracy. Further exploration of alternative approaches to EEG data processing in special populations is warranted.

## Consent

Written informed consent was obtained from the patient’s guardian/parent/next of kin for the publication of this report and any accompanying images’.

## Abbreviations

ADOS: Autism Diagnostic Observation Schedule; ASD: autism spectrum disorder; CARS2: Childhood Autism Rating Scale (second edition); CSS: Calibrated Severity Score; EEG: electroencephalogram; ERP: event-related potentials; ICA: independent component analysis; LOOCV: leave-one-out cross-validation; mMSE: modified multiscale entropy; SSD: sum of signed differences; SVM: support vector machine; TD: typically developing.

## Competing interests

The authors declare that they have no competing interests.

## Authors’ contributions

JE carried out the analysis, wrote codes to do the necessary calculations, performed the classification, and drafted the corresponding sections of the manuscript. AEL collected the data on which the results are reported, contributed to the introduction and methods section of the manuscript, and aided in the design of the ERP study. MB aided in the design of the classifier and the discussion of the results. SD aided in the design of the experiment, the design of the artifact removal and feature extraction process, and the discussion of results. All authors read and approved the final manuscript.
